# Short and Long-Term Impact of Lipectomy on Expression Profile of Hepatic Anabolic Genes in Rats: A High Fat and High Cholesterol Diet-Induced Obese Model

**DOI:** 10.1371/journal.pone.0108717

**Published:** 2014-09-29

**Authors:** Bey-Leei Ling, Chun-Tang Chiu, Hsiu-Chin Lu, Jin-Jin Lin, Chiung-Yin Kuo, Fen-Pi Chou

**Affiliations:** 1 Institute of Biochemistry and Biotechnology, College of Medicine, Chung Shan Medical University, Taichung City, Taiwan; 2 Department of Laboratory Medicine, China Medical University Hospital, Taichung City, Taiwan; 3 Institute of Biotechnology, HungKuang University, Taichung City, Taiwan; 4 Genomics Research Center, Academia Sinica, Taipei City, Taiwan; 5 Clinical Laboratory, Chung Shan Medical University Hospital, Taichung City, Taiwan; University of Catanzaro Magna Graecia, Italy

## Abstract

**Objective:**

To understand the molecular basis of the short and long-term effects of an immediate shortage of energy storage caused by lipectomy on expression profile of genes involved in lipid and carbohydrate metabolism in high fat and high cholesterol diet-induced obese rats.

**Methods:**

The hepatic mRNA levels of enzymes, regulator and transcription factors involved in glucose and lipid metabolism were analyzed by quantitative real time polymerase chain reaction (RT-qPCR) ten days and eight weeks after lipectomy in obese rats. Body and liver weights and serum biochemical parameters, adiponectin, leptin and insulin were determined.

**Results:**

No significant difference was observed on the food intake between the lipectomized and sham-operated groups during the experimental period. Ten days after the operation, the lipectomized animals showed significant higher triacylglycerol, glucose and insulin levels, a lower adiponectin concentration than the sham-operated rats, along with significant higher hepatic mRNA levels of hepatocyte nuclear factor 4α (HNF4α) and the enzymes involved in lipogenesis, sterol biosynthesis and gluconeogenesis. The results of immunohistochemical (IHC) analysis also confirmed increased levels of lipogenic enzymes in the liver of lipectomized versus sham-operated animals. The lipectomized group had a significantly lower adiponectin/leptin ratio that was positively correlated to the level of LDL (r = 0.823, P<0.05) and negatively to glucose and insulin (r = −0.821 and −0.892 respectively, P<0.05). Eight weeks after the operation, the lipectomized animals revealed significant higher body and liver weights, weight gain, liver to body weight ratio, hepatic triacylglycerol and serum insulin level.

**Conclusions:**

In response to lipectomy a short term enhancement of the expression of hepatic anabolic genes involved in lipid and carbohydrate metabolism was triggered that might eventually lead to the final extra weight gain. These metabolic changes could be the results of reduced circulating adiponectin that further influences the functions of insulin and hepatic HNF4α.

## Introduction

Obesity and body fat deposition are well recognized to be associated with many diseases, such as Type II diabetes, hypertension, coronary artery disease, and cancer. A life style of overeating or under-activity for over an extended period of time that leads to excess energy intake over expenditure is the main cause of obesity. Diet-induced or exercise-induced weight loss is therefore considered to be the most effective means to reduce obesity and control weight. However, the esthetic surgery of liposuction has been considered another option for weight control.

Adipose tissue is the main fuel storage unit involved in the maintenance of energy homeostasis. The hypothesis of adipostat states that any alterations in the total amount of energy reserves will be compensated by alterations either in metabolism or in thermogenesis so that the body will revert to the ‘desired’ body weight [1], though the molecular mechanism still remains uncovered. A sudden shortage of energy storage caused by liposuction might lead to a reprogrammed metabolism in the liver which is the most important organ responsible for the regulation of energy metabolism. Clinical studies demonstrated that women undergoing liposuction regained fat within six months [2], [3]. This compensatory fat growth was confirmed in several animal models showing that lipectomy is accompanied by fat regain within a few weeks [4]–[7] probably due to feedback mechanisms triggered by immediate loss of body fat with a molecular basis that needs to be defined. Earlier clinical observations noted that some patients undergoing extensive surgical reduction of subcutaneous adipose tissue postoperatively had increased serum insulin and triglyceride levels [8]. Some patients who had had prior lipectomy subsequently developed severe or “morbid” obesity with non-insulin-dependent diabetes and dyslipidemia [9]. The development of hyperinsulinemia, hyperlipidemia and metabolic syndrome was also observed in animal studies [5], [6].

In order to understand the molecular basis of the impact of lipectomy on energy metabolism, we performed a surgical partial fat removal on high fat and high cholesterol-induced obese rats, and analyzed quantitatively the hepatic gene expression of enzymes, regulator and transcription factors involved in glucose and lipid metabolism along with serum biochemical parameters and hormones ten days and 8 weeks after the operation. To our knowledge this is the first study that looked into the short-term and long-term effect of immediate deficiency in energy storage on the metabolic gene profile.

## Materials and Methods

### Ethics Statement

All animal care and experimental procedures were carried out in strict accordance with the guidelines for the care and use of laboratory animals of Chung Shan Medical University, and approved by the Institutional Animal Care and Use Committee (Permit Number: 942). All surgery was performed under Zoletil anesthesia, and all efforts were made to minimize suffering.

### Animal treatment

**Figure 1:**
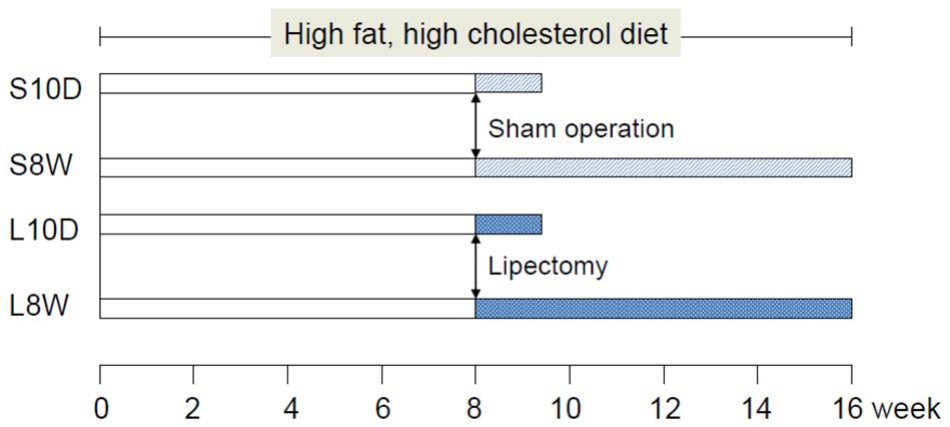
Experimental design and designation of group name.

Female Sprague-Dawley rats (Laboratory Animal Center, Hualien, Taiwan) weighing about 200 g were maintained in a temperature-controlled room (24°C) and illuminated for 12 hours daily. The experimental design and the designation of group name are shown in Fig. 1. The animals were on a normal diet or fed with a chow (Laboratory Rodent Diet 5001, PMI Nutrition International/Purina Mills LLC, MO, USA) supplemented with 2% cholesterol (Sigma-Aldrich, MO, USA) and 20% lard for 8 weeks to induce obesity and then all the way through the experiment. The chow and water were supplied ad libitum. The daily food intake was quantified twice weekly and calculated as mass of food consumed daily per rat. One half of the animals were surgically removed partial abdominal fat (1.5 to 2.5% of body weight), and the other half were sham-operated at 8 week. One half of both lipectomized and sham-operated rats (n = 8 for each groups) were sacrificed 10 days after the operation and designated as L10D and S10D groups, and the other half (n = 8 for each groups) were sacrificed 8 weeks later and designated as L8W and S8W groups. Blood and liver samples were collected at the time of sacrifice. The body and liver weight of all animals was registered. Serum was prepared and stored at −80°C until analysis. Part of liver tissue was fixed in formalin for pathological examination after stained with hematoxylin and eosin and for immunohistochemistry, and parts were used for RNA isolation and hepatic triacylglycerol (TG) determination.

### Analysis of hepatic TG

Liver lipids were extracted as described previously [10]. Briefly, liver (1.25 g) was homogenized with chloroform/methanol (v/v, 1∶2), and then added and thoroughly mixed with chloroform (1.25 ml) and distilled water (1.25 ml). After centrifugation (1500 g for 10 min), the lower clear organic phase solution was transferred into a new glass tube and then lyophilized. The lyophilized powder was dissolved in chloroform/methanol (v/v, 1∶2) and then analyzed for TG content by enzymatic colorimetric methods using a commercial kit (HUMAN, Germany).

### Analysis of biochemical markers and lipoproteins

The serum concentrations of total cholesterol, TG, glucose, and low and high density lipoproteins (LDL and HDL) were determined enzymatically with a Cobas Integra 400 auto analyzer (Roche, Switzerland).

### Analysis of leptin, insulin and adiponectin

Serum leptin, insulin and adiponectin concentrations were measure by ELISA kits for rat according to the procedures provided by the supplier (Millipore, MO, USA).

### RNA isolation

Total RNA was extracted from samples with Trizol reagent (Invitrogen, Leek, Netherlands) according to the manufacturer's instructions. RNA yields were quantified spectrophotometrically. RNA quality was examined by means of the A260/A280 ratio, and confirmed by RNA agarose gel electrophoresis to observe the integrity of 18S and 28S rRNAs. Reverse transcription was performed with a commercial kit (RevertAid First Strand cDNA Synthesis Kit; Fermentas, MD, USA) following the procedures provided by the manufacture.

### Quantitative real time polymerase chain reaction (RT-qPCR) array analysis

Custom RT2 Profiler PCR Array plates containing the 14 genes focused in the study and ribosomal protein, large, P1 (Rplp1) as the internal control were ordered and obtained from SABiosciences (MD, USA). Quantitative real-time PCR was performed using Applied Biosystems 7700 sequence detection system under conditions suggested by SABiosciences Co. for 96-well array plate.

### Immunohistochemical analysis

Immunohistochemical analysis was performed on formalin-fixed Paraffin-embedded liver tissue. Sections (3 um) on coated slides were deparaffinized and rehydrated then subjected to antigen retrieval by autoclave or microwave in alkaline buffer pH 9 (antigen Retrieval AR10, BioGeneX) for 10 minutes. After treated with H_2_O_2_ to block the endogenous peroxidase activity, the sections were incubated with diluted primary antibodies as indicated by the manufacturer (Biorbyt, UK) at room temperature for one hour, followed by staining with Super Sensitive Polymer-HRP Detection System (BioGenex, CA, USA), counter-staining with Mayer's hematoxylin and mounted in glycerin. Sections were examined by a pathologist, and the staining was semiquantitatively scored as 5 grades.

### Statistical analysis

All data were presented as mean±SE. Two-way ANOVA was used to test a significant effect of lipectomy, time and interaction between two factors on parameters with a *post-hoc* analysis of Holm Sidak test that was always performed for all pairwise comparisons. One-way repeated measures ANOVA (RMANOVA) was used to test a significance between different time points using a *post-hoc* analysis of Student-Newman-Keuls Method. One-way ANOVA was used to analyze the significance among four groups and student t-test was used between two groups at each time point. Correlation between two parameters was calculated by Pearson Product Moment Correlation. P<0.05 was considered statistically significant (Sigma-Stat 3.5, San Rafael, CA, USA).

## Results

### Short and long-term effect of lipectomy on daily food intake, body weight, liver weight, and serum glucose, lipids, insulin, leptin and adiponectin

**Figure 2:**
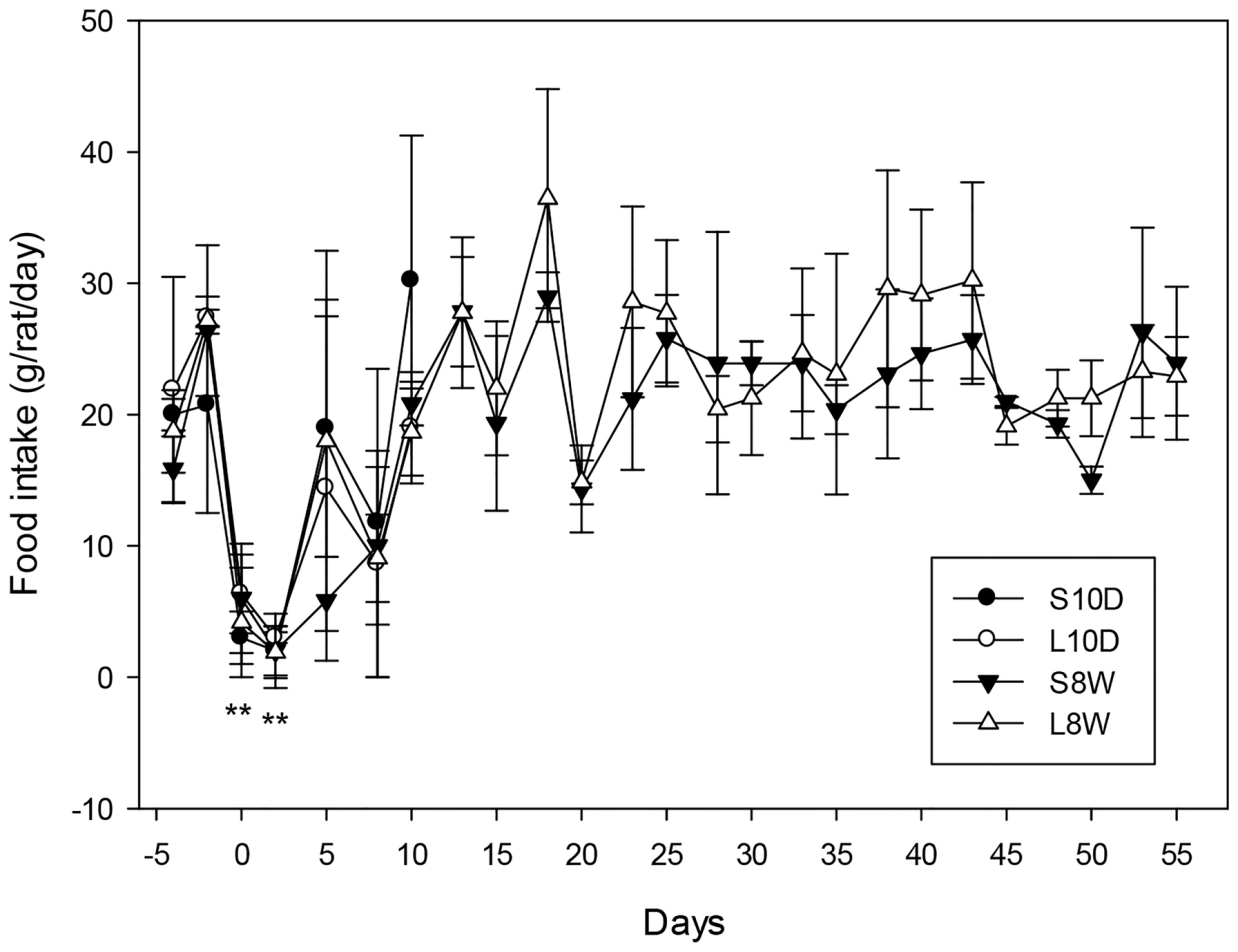
Changes in daily food intake. The food intake was quantified every two or three days and calculated as mass of food consumed daily per rat 4 days before and 55 days after the operation (day 0). * p<0.05, ** p<0.01: significantly lower than the other time points as analyzed by One-way RMANOVA. One-way ANOVA was used to analyze the significance among four groups and student t-test was used between two groups at each time point.

**Figure 3:**
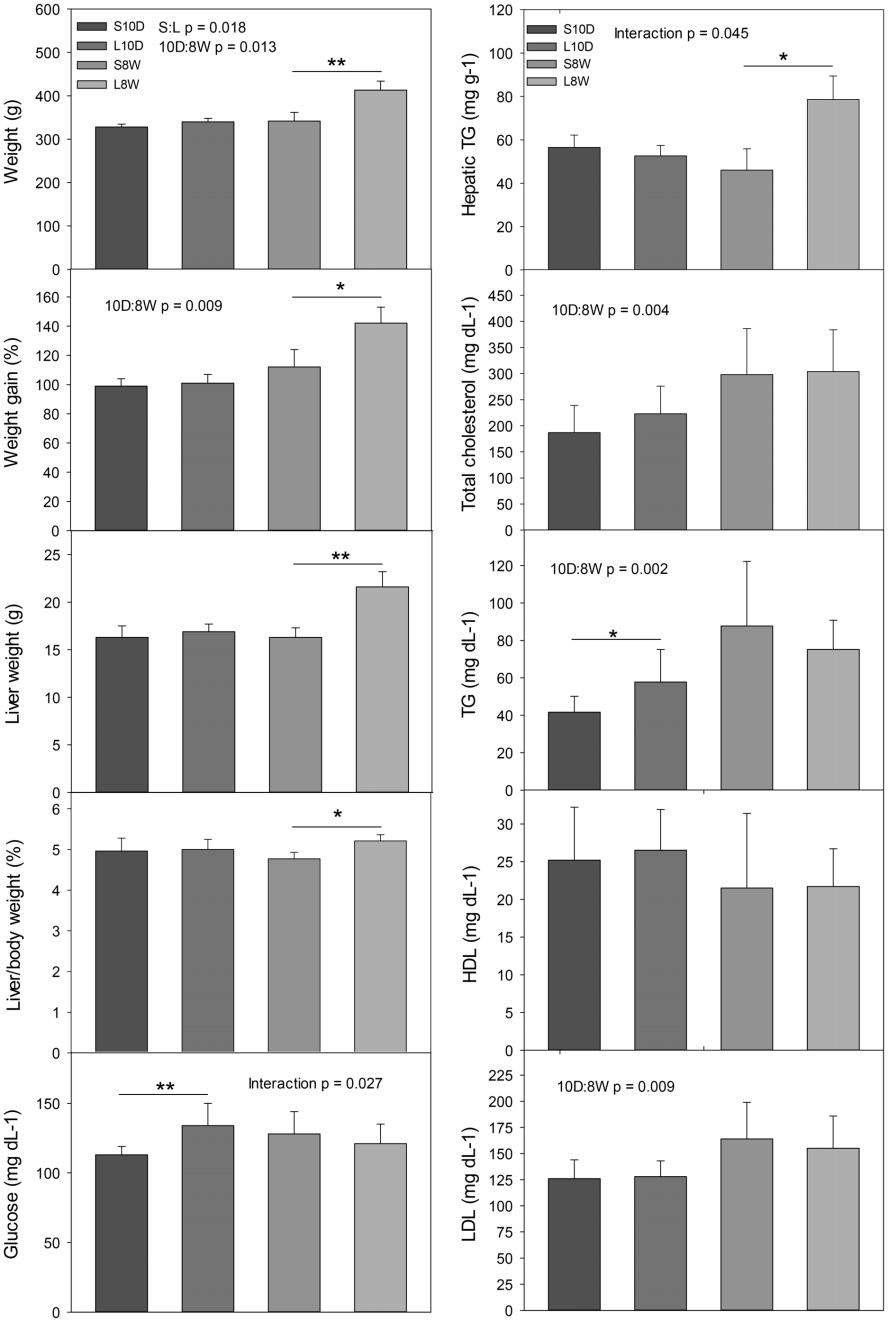
Short and long-term effects of lipectomy on physiological and serum biochemical parameters. Blood and liver samples were collected at the time of sacrifice. The body and liver weight of all animals was registered. Serum was prepared and analyzed for biochemical parameters and hormones. The significant p value of Two-way ANOVA analysis for an effect of lipectomy and sham operation (S:L), 10 day and 8 week time point (10D:8W) and interaction between two factors on a parameter was shown. * p<0.05, ** p<0.01: Two-way ANOVA Holm Sidak *posthoc* analysis of significant difference.

The average daily food intake presented in Fig. 2 showed that the surgical process caused an acute drop in food consumption that was returned to normal ranges in 10 days. After that, no significant difference on food intake was observed between S8W and L8W groups. Ten days after the operation, the lipectomized and sham-operated animals exhibited no significant differences in the average body and liver weights and in the serum levels of total cholesterol, LDL and HDL (Fig. 3, L10D and S10D groups). However, the lipectomized group showed a significant higher level of TG, glucose and insulin (Fig. 3 and Table 1), and a lower concentration of adiponectin (Table 1) as compared to those of the time-matched sham-operated group at 10-day. Although leptin concentration was not significantly different between these two groups, the lipectomized group had significantly lower adiponectin/leptin ratio (Table 1) that was positively correlated to the level of LDL (r = 0.823, p<0.05) and negatively to glucose and insulin (r = −0.821 and −0.892 respectively, p<0.05) (Table 1 footnote). The animals sacrificed 8 weeks after the operation showed significant higher body and liver weights, weight gain and serum insulin level in the lipectomized group than in the time-matched sham-operated group (Fig. 3 and Table 1, L8W and S8W groups). On the contrary, the serum level of glucose and lipids (TG, cholesterol, LDL and HDL) were similar between these two groups. The adiponectin/leptin ratio of L8W group was negatively correlated to insulin (r = −0.892, p<0.05) (Table 1 footnote), but was not significantly different from that of S8W.

**Table 1:** Short and long-term effects of lipectomy on hormones in obese rats.

	S10D	L10D	S8W	L8W	2 way ANOVA analysis
Adiponectin (µg ml-1)	18.86±8.26	10.45±3.67*	18.61±4.06	14.39±4.80	S:L p = 0.011
Leptin (ng ml-1)	2.14±1.53	3.75±1.84	10.2±5.10	9.72±4.50	10D:8W p<0.001
Insulin (ng ml-1)	0.44±0.20	0.74±0.31*	1.02±0.66	1.59±0.48*	S:L p = 0.035 10D:8W p = 0.001
Adiponectin/leptin ratio	13.78±4.10	3.34±0.63* a	1.62±0.26	1.71±0.31^b^	S:L p = 0.01 10D:8W p = 0.026

* p<0.05, Two-way ANOVA Holm Sidak *posthoc* analysis of significant difference between the lipectomized and the time-matched sham-operated groups.

a positively correlated to the level of LDL (r = 0.823, p<0.05) and negatively to glucose and insulin (r = −0.821 and −0.892 respectively, p<0.05) as calculated by Pearson Product Moment Correlation.

b negatively correlated to insulin (r = −0.892, p<0.05) as calculated by Pearson Product Moment Correlation.

### Excess lipid accumulation in the liver eight weeks after lipectomy

**Figure 4:**
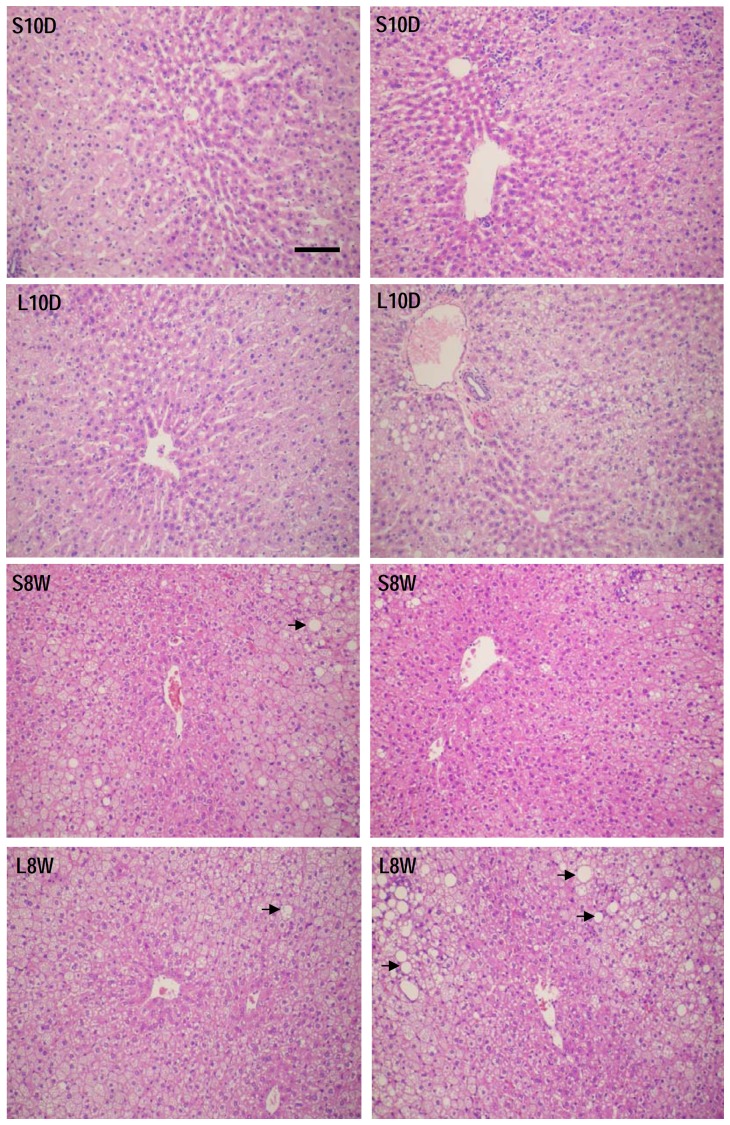
Hematoxylin and eosin stain of liver sections. Liver tissues were collected from lipectomized (L10D and L8W) and sham-operated (S10D and S8W) rats and stained with Hematoxylin and eosin for pathological observation. Two representative animals from each group were shown. Arrow: lipid vacuole. The scale bar represents 100 µm.

The morphological examination of the liver sections illustrated similar hepatic morphology between lipectomized and sham-operated rats 10 days after the surgery (Fig. 4). The livers obtained from the animals sacrificed 8 weeks later appeared to have more lipid vacuoles in the hepatocytes (Fig. 4, arrows). Two-way ANOVA analysis of the data of liver to body weight ratio and hepatic TG content Showed that these two parameters were significantly higher in L8W group (Fig. 3), suggesting that these animals had more lipid accumulation in the liver 8 weeks after the surgery.

### Increased mRNA expression of hepatic anabolic genes in lipectomized rats 10 days after operation

Fat accumulation in the liver could result from a deregulation of lipid synthesis, lipid oxidation, gluconeogenesis and energy expenditure. We analyzed the transcript levels of hepatic metabolic genes by RT-qPCR 10 days and 8 weeks after the operation. Two-way ANOVA analysis revealed a operation effect on the transcript levels of acetyl-CoA carboxylase 1 (ACC1), fatty acid synthase (FAS), 3-hydroxy-3-methyl-glutaryl-CoA reductase (HMG), fructose-1,6-bisphosphatase 1 (FBP1), and hormone-sensitive lipase (HSL) (Table 2). A time effect was observed for the expression of FAS, HMG, FBP1, Glycogen synthase kinase 3β (GSK3β), uncoupling protein 2 (UCP2), and HSL. *Post-hoc* analysis for all pairwise comparisons showed that the mRNA levels of lipogenic enzymes (ACC1 and FAS) and sterol biosynthetic enzyme (HMG) were significantly higher in the livers obtained from the lipectomized rats than those from the sham-operated ones 10 days after the surgery, and were similar between two groups at 8-week (Table 2). The enzymes involved in the fatty acid oxidation (acetyl-CoA carboxylase 2, ACC2 and carnitine palmitoyltransferase 1A, CPT1A) were not significantly different between the lipectomized and the sham-operated animals at both 10-day and 8-week time points. Lipectomy also led to higher mRNA levels of gluconeogenic enzymes (FBP1 and glucose 6-phosphatase, G6Pase) at 10-day, but not 8-week. GSK3β which regulates glycogen synthase and UCP2 which dissipates energy as heat were not affected by fat removal at neither time points. The transcript level of HSL which is activated when the body needs to mobilize energy stores was increased in the lipectomized rats as compared to the sham-operated ones at 10-day. On the contrary, glycerol kinase (GK) which catalyzes TG synthesis was inhibited by lipectomy at 10-day.

**Table 2:** Short and long-term effects of lipectomy on hepatic transcript levels of 14 genes in obese rats.

	S10D ΔCt^a^	L10D ΔCt	L10D/S10D Fold change^b^	S8W ΔCt	L8W ΔCt	L8W/S8W Fold change	2 way ANOVA analysis
Metabolic gene							
ACC1	6.32±0.79	4.78±0.89	2.91*	5.51±0.37	5.15±0.84	1.28	S:L p = 0.007
FAS	5.09±0.61	3.31±1.75	3.43*	3.00±0.45	2.59±1.31	1.33	S:L p = 0.039 10D:8W p = 0.010
HMG	5.03±0.53	3.99±0.72	2.06*	5.15±0.43	4.91±0.28	1.18	S:L p = 0.008 10D:8W p = 0.027
ACC2	7.04±0.71	6.02±1.69	2.03	7.04±0.41	6.15±0.95	1.85	
CPT1A	2.82±0.83	2.84±0.51	0.99	3.66±0.95	3.10±0.40	1.47	
FBP1	−0.24±0.11	−0.68±0.32	1.36*	0.04±0.13	−0.19±0.15	1.17	S:L p = 0.022 10D:8W p = 0.011
G6Pase	1.93±0.34	1.06±0.38	1.83*	1.75±0.38	1.90±0.62	0.90	
GSK3β	4.78±0.38	5.02±0.43	0.85	5.55±0.32	5.44±0.15	1.08	10D:8W p<0.001
UCP2	3.83±0.41	3.47±0.71	1.28	4.49±0.77	4.14±0.43	1.27	10D:8W p = 0.015
HSL	6.98±0.21	5.76±1.02	2.33*	7.25±0.53	6.91±0.30	1.27	S:L p = 0.008 10D:8W p = 0.015
GK	2.05±0.39	2.82±0.24	0.59**	2.83±0.62	2.70±0.20	1.09	Interaction p = 0.013
Transcription factor and regulator							
HNF4α	2.29±0.39	1.41±0.35	1.84**	2.12±0.50	2.11±0.15	1.01	S:L p = 0.010 Interaction p = 0.011
AMPKα	6.45±0.85	6.07±0.82	1.30	7.10±1.85	6.69±0.42	1.33	
SREBP1	3.00±0.95	2.75±1.42	1.19	2.42±0.55	2.17±0.77	1.19	

* p<0.05,

** p<0.01, Two-way ANOVA Holm Sidak *posthoc* analysis of significant difference between the lipectomized and the time-matched sham-operated groups.

a ΔCt: Ct values normalized with respective Rplp1 Cts, where Ct is the threshold cycle of RT-qPCR.

b 2^−(L10DΔCt - S10DΔCt)^.

### Increased mRNA expression of HNF4α in lipectomized rats 10 days after operation

To further identify which factor might be responsible for the hepatic response to fat removal, we analyzed the expression levels of HNF4α, AMP-activated protein kinase α (AMPKα) and sterol regulatory element-binding protein 1 (SREBP1). HNF4α is a nuclear transcription factor that regulates various genes involved in glucose, fatty acid, amino acid, and cholesterol metabolism, as well as blood coagulation and hepatic development and differentiation [11]. AMPK is considered as a cellular energy sensor by limiting anabolic pathways and by facilitating catabolic pathways [12]. SREBP-1 is a transcription factor that has been implicated in the effect of insulin on the expression of key genes of lipid and glucose metabolism [13]. Among these three genes, only the expression of HNF4α was enhanced by fat removal 10 days after the surgery (Table 2). The difference in HNF4α level between the lipectomized and the sham-operated groups was lost in the long term.

### Increased protein level of hepatic lipogenic genes in lipectomized rats 10 days after operation

**Figure 5:**
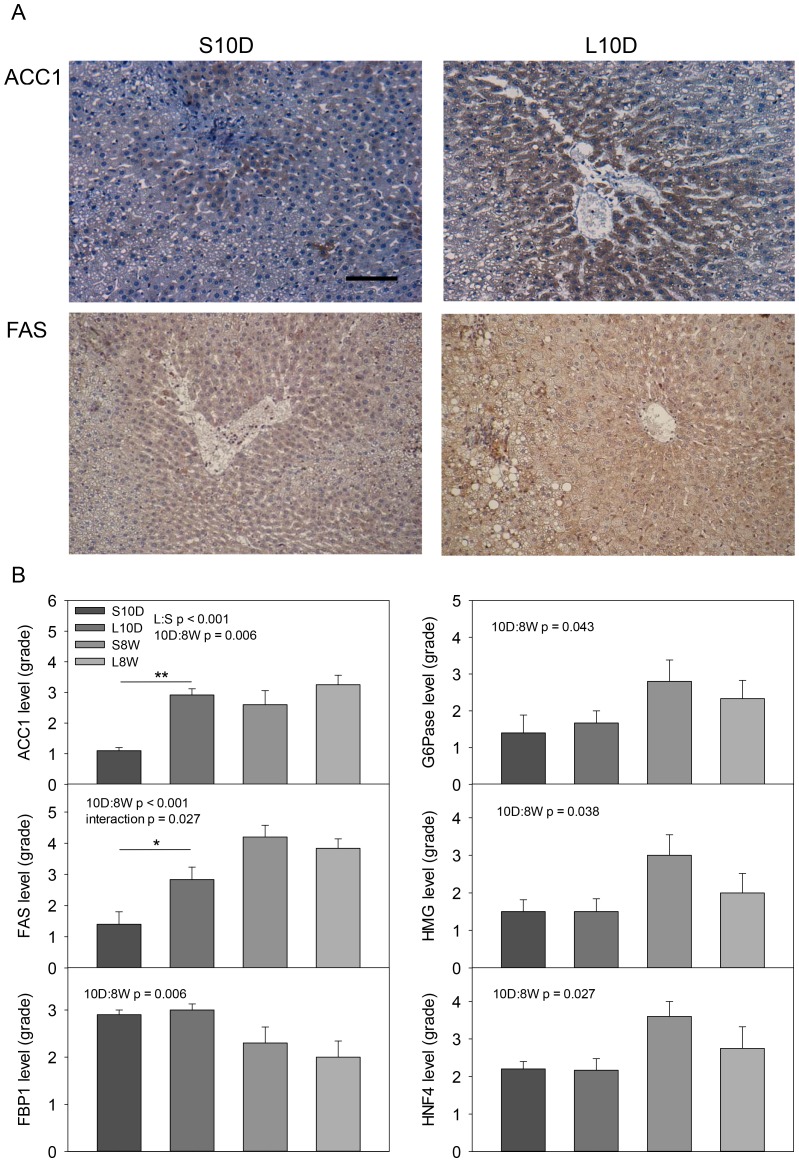
Short and long-term effects of lipectomy on hepatic protein levels in obese rats. Immunohistochemical analysis was performed on formalin-fixed Paraffin-embedded liver tissues collected from lipectomized (L10D and L8W) and sham-operated (S10D and S8W) rats. (A) Representatives of liver sections detected with ACC1 or FAS primary antibody on S10D and L10D samples. (B) Quantitative data of the IHC results scored as 5 grades. The significant p value of Two-way ANOVA analysis for an effect of lipectomy and sham operation (S:L), 10 day and 8 week time point (10D:8W) and interaction between two factors on a parameter was shown. * p<0.05, ** p<0.01: Two-way ANOVA Holm Sidak *posthoc* analysis of significant difference. The scale bar represents 100 µm.

In order to examine the protein levels of the lipectomy-enhanced anabolic genes (ACC1, FAS, FBP1, G6Pase and HMG), liver sections were subjected to immunohistochemical analysis with respective primary antibody. Among the 5 genes, the lipogenic enzymes (ACC1 and FAS) were significantly more abundant in the livers obtained from the lipectomized rats than those from the sham-operated ones 10 days after the surgery (Fig. 5A and B), while the other three enzymes were similar in lipectomized versus sham-operated mice. There was no significant difference in the protein level for all genes at 8 week time point (Fig. 5B). However, Two-way ANOVA analysis revealed a time effect on the protein level of all five genes, showing that the 8-week samples have more enzymes than the 10-day tissues except FBP1 (Fig. 5B).

## Discussion

By using lipectomy to create a condition of energy shortage and then observing the expression of hepatic genes at 10-day as a short term response and at 8-week as a long-term outcome, we demonstrated that the hepatic anabolic pathways were enhanced in response to fat removal. On the basis that no significant difference was observed on the food intake between the lipectomized and the sham-operated groups, the results indicated that an immediate loss of fat storage in obese rats might trigger a response that tends to increase energy accumulation that eventually leads to excess weight regain to an extent even greater than the ones without lipectomy.

In this study the animals underwent lipectomy weighed heavier than the sham-operated ones 8 weeks after the surgery, and had more lipid accumulation in the liver concurrently. However, the serum concentrations of glucose and lipids showed no difference between these two groups, indicating the influence of lipectomy on energy metabolism have dissipated at this time point. This was confirmed by the gene expression analysis revealing comparable transcript levels for all the tested genes between these two groups. The animals sacrificed 10 days after the surgery provided evidences for the feedback consequence of lipectomy. The liver of these animals possessed higher mRNA levels of lipogenic (ACC1, FAS), sterol synthetic (HMG) and gluconeogenic (FBP1, G6Pase) enzymes than the sham-operated ones, suggesting that lipectomy stimulated the hepatic anabolic pathways with an intention to restore the loss of energy storage. Excess weight gain and lipid accumulation could also be a result of energy conservation. However, the genes involved in β-oxidation of fatty acid, and energy expenditure were not affected by lipectomy. Furthermore, AMPK which is known to activate fatty acid oxidation and glycolysis, and to inhibit fatty acid, TG, cholesterol and glycogen synthesis and gluconeogenesis [14] was also unaffected. A surprising result is that the expression of HSL was induced by fat removal, while de novo synthesis of fatty acid was enhanced at the same time. We speculate that these might lead to excess accumulation of free fatty acids that has been shown to cause lipotoxicity and inflammation of the liver [15]. Taken together, the data indicate that lipectomy tend to enhance the anabolic pathways, but leave the catabolic pathways unaffected. However, the results of IHC supported only the enhancement of lipogenic pathway by lipectomy but not gluconeogenesis. Even so, we cannot exclude a possible impact on gluconeogenic pathway, because the animals were on high fat diet and the samples were collected 10 days after the surgery at a fixed time point that the food intake had just resumed to normal range (Fig. 2). It is likely that the influence of fat removal on the expression time frame of lipogenic genes was more direct and prior to that of gluconeogenic genes. Although both transcription factors HNF4α and SREBP1 are known to regulate expression of genes involved in lipogenesis and sterogenesis [16], [17], only the transcript level of HNF4α was enhanced by lipectomy. HNF4α has a large number of target genes, many of which are involved in liver functions such as carbohydrate metabolism (glucose-6-phosphatase, glucokinase regulatory protein) and lipogenesis (fatty acid synthase, stearoyl-CoA desaturase) [18], [19]. HNF4α has been demonstrated to activate insulin gene expression indirectly via inducing the level of hepatic nuclear factor 1 [20], [21] and also directly through binding to a cis element of insulin promoter [22], and is recognized as a cause of maturity onset diabetes of the young, subtype 1. On the other hand, insulin activates the transcriptional activity of HNF4 via forkhead in human rhabdomyosarcoma (FKHR) as a signal-regulated transcriptional inhibitor [23]. In this study the serum concentration of insulin was significant higher in the lipectomized rats than in the sham-operated animals no matter analyzed at 10-day or 8-week time points. This elevated circulating insulin could enhance the hepatic HNF4α function. However, it remained to be clarified whether HNF4α takes part in the regulation of the enhanced anabolic pathways or it is a consequence of the elevated circulating insulin level in lipectomized animals. Furthermore, since only the transcripts of transcription factors were examined in this study, we cannot decisively rule out the participation of AMPKα and SREBP1.

Both adiponectin and leptin are adipokines that regulate glucose and lipid metabolism, however only the former is exclusively secreted from adipose tissue, and also from the placenta in pregnancy [24]. Adiponectin has been reported to repress hepatic gluconeogenesis, an important mechanism to improve insulin sensitivity [25]. A low level of adiponectin is an independent risk factor for developing metabolic syndrome [26] and diabetes mellitus [27]. The study of adiponectin knockout mice demonstrated that adiponectin plays an important role in regulating the expression of rate-limiting enzymes in several important glucose and lipid metabolic pathways [28]. Animal studies indicated that adiponectin reduced level of plasma TG, exerting a protective effect on triacylglycerol metabolism [29]. In this study fat removal led to a reduced level of circulating adiponectin that might release its repression on hepatic gluconeogenesis and lose its positive effects on TG metabolism and insulin sensitivity, as evidenced by the higher plasma levels of glucose, TG and insulin in these animals. Leptin, on the other hand, was not significantly affected by lipectomy because of high interindividual variation. Although it circulates at levels directly proportional to body fat [30], considerable interindividual variation was observed in overweight and obese men [31]. These observations indicate that the response induced by lipectomy, a surgery that causes an immediate deficiency of energy storage, is mediated via mechanisms distinct from those induced by an energy-restricted diet that showed an acute proportional reduction in fasting leptin [32].

In this study we observed a lower adiponectin/leptin ratio that, but not adiponectin or leptin alone, is negatively correlated to glucose and insulin concentrations 10 days after lipectomy, and a sustained higher insulin level throughout the experiment. This observation was consistent with the outcomes of recent studies suggesting that the adiponectin/leptin ratio was a more sensitive and reliable marker of insulin resistance in relevance to adiponectin or leptin alone in type 2 diabetes and in subjects without hyperglycemia [31], [33]–[35], and it is related to the metabolic risk susceptibility in men [31]. Men with high adiponectin/leptin ratios have better triacylglycerol profile and insulin sensitivity. These results further emphases that it is the adiponectin that plays the essential role in current model, although both adiponectin and leptin might make contribution to hyperinsulinemia, hyperlipidemia. Therefore, an immediate shortage of energy storage might cause deregulation of adipokine secretion that in turn influences the level of insulin and hepatic gene expression.

**Figure 6:**
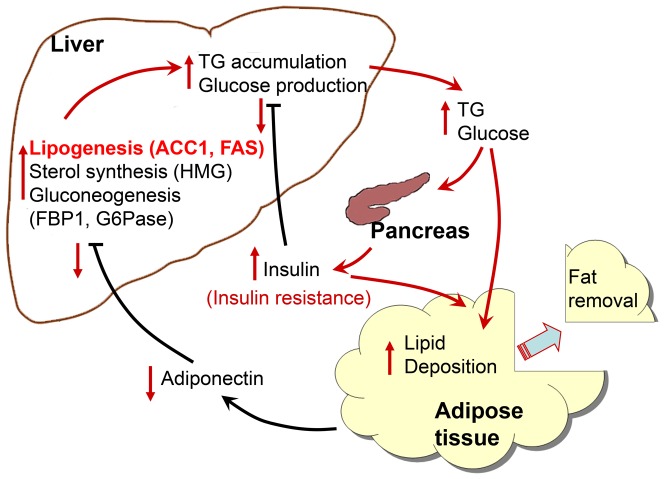
A proposed scheme of the molecular basis of the impact of lipectomy in obese rats. Fat removal in obese rats caused a reduced level of circulating adiponectin that would release its inhibition on the hepatic anabolic pathways, especially lipogenesis, and subsequently enhanced TG and glucose productions. The increase level of blood glucose stimulated the secretion of insulin and augmented insulin resistance in the long run that would further remove the inhibitory action of insulin on glucose production. These short term metabolic changes in response to the condition of an immediate shortage of energy storage ultimately led to long-term excess lipid deposition. Red arrows represent the events in response to fat removal.

In this study we observed that the expression of genes involved in hepatic anabolic pathways, especially lipogenesis, were enhanced in response to the condition of an immediate shortage of energy storage that was achieved by removing a portion of adipose tissue. It is highly speculated that these short term metabolic changes are the result of a reduced level of circulating adiponectin, and are the causes of long-term excess weight regain and lipid accumulation in the liver, as proposed in the scheme shown in Fig. 6. The serum concentration of insulin was remained higher in the lipectomized rats throughout the experiment, an effect that might lead to metabolic disturbance and insulin resistance.

## References

[1] Frayling TM, Timpson NJ, Weedon MN, Zeggini E, Freathy RM, et al (2007) A common variant in the FTO gene is associated with body mass index and predisposes to childhood and adult obesity. Science 316: 889–894. 17434869 10.1126/science.1141634 PMC2646098

[2] Hernandez TL, Kittelson JM, Law CK, Ketch LL, Stob NR, et al (2011) Fat redistribution following suction lipectomy: defense of body fat and patterns of restoration. Obesity 19: 1388–1395. 21475140 10.1038/oby.2011.64

[3] Benatti F, Solis M, Artioli G, Montag E, Painelli V, et al (2012) Liposuction induces a compensatory increase of visceral fat which is effectively counteracted by physical activity: a randomized trial. J Clin Endocrinol Metab 97: 2388–2395. 22539589 10.1210/jc.2012-1012

[4] Mauer MM, Bartness TJ (1997) Fat pad-specific compensatory mass increases after varying degrees of lipectomy in Siberian hamsters. Am J Physiol 273: R2117–R2123. 9435669 10.1152/ajpregu.1997.273.6.R2117

[5] Liszka TG, Dellon AL, Im M, Angel MF, Plotnick L (1998) Effect of lipectomy on growth and development of hyperinsulinemia and hyperlipidemia in the Zucker rat. Plast Reconstr Surg 102: 1122–1127. 9734431 10.1097/00006534-199809040-00031

[6] Weber RV, Buckley MC, Fried SK, Kral JG (2000) Subcutaneous lipectomy causes a metabolic syndrome in hamsters. Am J Physiol Regul Integr Comp Physiol 279: R936–R943. 10956251 10.1152/ajpregu.2000.279.3.R936

[7] Hausman DB, Lu J, Ryan DH, Flatt WP, Harris RB (2004) Compensatory growth of adipose tissue after partial lipectomy: involvement of serum factors. Exp Biol Med 229: 512–520. 10.1177/153537020422900609 15169970

[8] Kral JG, Sjöström LV (1975) Surgical reduction of adipose tissue hypercellularity. Recent Adv Obes Res 1: 327–330.

[9] Kral JG (1988) Surgical treatment of regional adiposity. Acta Med Scand Suppl 723: 225–231. 3164970

[10] Folch J, Lees M, Sloane SGH (1957) A simple method for the isolation and purification of total lipids from animal tissues. J Biol Chem 226: 497–509. 13428781

[11] Martinez-Jimenez CP, Kyrmizi I, Cardot P, Gonzalez FJ, Talianidis I (2010) Hepatocyte nuclear factor 4 coordinates a transcription factor network regulating hepatic fatty acid metabolism. Mol Cell Biol 30: 565–577. 19933841 10.1128/MCB.00927-09 PMC2812226

[12] Viollet B, Guigas B, Leclerc J, Hébrard S, Lantier L, et al (2009) AMP-activated protein kinase in the regulation of hepatic energy metabolism: from physiology to therapeutic perspectives. Acta Physiol 196: 81–98. 10.1111/j.1748-1716.2009.01970.x PMC2956117 19245656

[13] Osborne TF (2000) Sterol regulatory element-binding proteins (SREBPs): key regulators of nutritional homeostasis and insulin action. J Biol Chem 275: 32379–32382. 10934219 10.1074/jbc.R000017200

[14] Ruderman N, Prentki M (2004) AMP kinase and malonyl-CoA: targets for therapy of the metabolic syndrome. Nat Rev Drug Discov 3: 340–351. 15060529 10.1038/nrd1344

[15] Savary S, Trompier D, Andréoletti P, Le Borgne F, Demarquoy J, et al (2012) Fatty acids-induced lipotoxicity and inflammation. Curr Drug Metab 13: 1358–1370. 22978392 10.2174/138920012803762729

[16] Eberlé D, Hegarty B, Bossard P, Ferré P, Foufelle F (2004) SREBP transcription factors: master regulators of lipid homeostasis. Biochimie 86: 839–848. 15589694 10.1016/j.biochi.2004.09.018

[17] Yin L, Ma H, Ge X, Edwards PA, Zhang Y (2011) Hepatic hepatocyte nuclear factor 4a is essential for maintaining triglyceride and cholesterol homeostasis. Arterioscler Thromb Vasc Biol 31: 328–336. 21071704 10.1161/ATVBAHA.110.217828 PMC3079249

[18] Odom DT, Zizlsperger N, Gordon DB, Bell GW, Rinaldi NJ, et al (2004) Control of pancreas and liver gene expression by HNF transcription factors. Science 303: 1378–1381. 14988562 10.1126/science.1089769 PMC3012624

[19] Adamson AW, Suchankova G, Rufo C, Nakamura MT, Teran-Garcia M, et al (2006) Hepatocyte nuclear factor-4alpha contributes to carbohydrate-induced transcriptional activation of hepatic fatty acid synthase. Biochem J 399: 285–295. 16800817 10.1042/BJ20060659 PMC1609920

[20] Boj SF, Parrizas M, Maestro MA, Ferrer J (2001) A transcription factor regulatory circuit in differentiated pancreatic cells. Proc Natl Acad Sci USA 98: 14481–14486. 11717395 10.1073/pnas.241349398 PMC64707

[21] Thomas H, Jaschkowitz K, Bulman M, Frayling TM, Mitchell SM, et al (2001) A distant upstream promoter of the HNF-4alpha gene connects the transcription factors involved in maturity-onset diabetes of the young. Hum Mol Genet 10: 2089–2097. 11590126 10.1093/hmg/10.19.2089

[22] Bartoov-Shifman R, Hertz R, Wang H, Wollheim CB, Bar-Tana J, et al (2002) Activation of the insulin gene promoter through a direct effect of hepatocyte nuclear factor 4. J Biol Chem 277: 25914–25919. 11994285 10.1074/jbc.M201582200

[23] Hirota K, Daitoku H, Matsuzaki H, Araya N, Yamagata K, et al (2003) Hepatocyte nuclear factor-4 is a novel downstream target of insulin via FKHR as a signal-regulated transcriptional inhibitor. J Biol Chem 278: 13056–13060. 12519792 10.1074/jbc.C200553200

[24] Chen J, Tan B, Karteris E, Zervou S, Digby J, et al (2006) Secretion of adiponectin by human placenta: differential modulation of adiponectin and its receptors by cytokines. Diabetologia 49: 1292–1302. 16570162 10.1007/s00125-006-0194-7

[25] Combs TP, Berg AH, Obici S, Scherer PE, Rossetti L (2001) Endogenous glucose production is inhibited by the adipose-derived protein Acrp30. J Clin Invest 108: 1875–1881. 11748271 10.1172/JCI14120 PMC209474

[26] Renaldi O, Pramono B, Sinorita H, Purnomo LB, Asdie RH, et al (2009) Hypoadiponectinemia: a risk factor for metabolic syndrome. Acta Med Indones 41: 20–24. 19258676

[27] Hara K, Yamauchi T, Kadowaki T (2005) Adiponectin: an adipokine linking adipocytes and type 2 diabetes in humans. Curr Diab Rep 5: 136–140. 15794918 10.1007/s11892-005-0041-0

[28] Liu Q, Yuan B, Lo KA, Patterson HC, Sun Y, et al (2012) Adiponectin regulates expression of hepatic genes critical for glucose and lipid metabolism. Proc Natl Acad Sci USA 109: 14568–14573. 22904186 10.1073/pnas.1211611109 PMC3437840

[29] Qiao L, Zou C, van der Westhuyzen DR, Shao J (2008) Adiponectin reduces plasma triglyceride by increasing VLDL triglyceride catabolism. Diabetes 57: 1824–1833. 18375436 10.2337/db07-0435 PMC2453618

[30] Brennan AM, Mantzoros CS (2006) Drug insight: the role of leptin in human physiology and pathophysiology-emerging clinical applications. Nat Clin Pract Endocrinol Metab 2: 318–327. 16932309 10.1038/ncpendmet0196

[31] Vega GL, Grundy SM (2013) Metabolic risk susceptibility in men is partially related to adiponectin/leptin ratio. J Obes 409679 doi: 10.1155/2013/409679 10.1155/2013/409679 PMC3606776 23533722

[32] Mars M, de Graaf C, de Groot CP, van Rossum CT, Kok FJ (2006) Fasting leptin and appetite responses induced by a 4-day 65%-energy-restricted diet. Int J Obes 30: 122–128. 10.1038/sj.ijo.0803070 16158086

[33] Inoue M, Maehata E, Yano M, Taniyama M, Suzuki S (2005) Correlation between the adiponectin-leptin ratio and parameters of insulin resistance in patients with type 2 diabetes. Metabolism 54: 281–286. 15736103 10.1016/j.metabol.2004.09.006

[34] Inoue M, Yano M, Yamakado M, Maehata E, Suzuki S (2006) Relationship between the adiponectin-leptin ratio and parameters of insulin resistance in subjects without hyperglycemia. Metabolism 55: 1248–1254. 16919546 10.1016/j.metabol.2006.05.010

[35] Zaletel J, Barlovic DP, Prezelj J (2010) Adiponectin-leptin ratio: a useful estimate of insulin resistance in patients with Type 2 diabetes. J Endocrinol Invest 33: 514–518. 20142631 10.1007/BF03346639

